# Supplementary material for: Risk factors of early mortality among COVID-19 deceased patients in Addis Ababa COVID-19 care centers, Ethiopia
Source: PLoS One. 2022 Sep 27;17(9):e0275131. doi: 10.1371/journal.pone.0275131 (PMC9514640; doi:10.1371/journal.pone.0275131)
Supplement: Attachment — Submitted filename: PLOSone COVID.pdf [file pone.0275131.s002.pdf]

PLOS ONE

Risk Factors of early mortality among COVID-19 deceased patients in Addis Ababa COVID-19 Care Centers, Ethiopia

--Manuscript Draft--

**Manuscript Number:**	PONE-D-22-10705
**Article Type:**	Research Article
**Full Title:**	Risk Factors of early mortality among COVID-19 deceased patients in Addis Ababa COVID-19 Care Centers, Ethiopia
**Short Title:**	Risk factors of early mortality among COVID-19 deceased patients
**Corresponding Author:**	Taye Ashine Mezgebu, MSc<br>Wachemo University<br>Hosanna, ETHIOPIA
**Keywords:**	Intensive care unit, early mortality, COVID-19, risk factors, Ethiopia
**Abstract:**	Background: Severe acute respiratory syndrome coronavirus-2 is a global health care problem with high mortality. Despite early mortality seeming alarming, data regarding factors that lead to increased early mortality of COVID 19 patients is not well-documented yet. The objective of this study was to identify the risk factors of early mortality in patients with confirmed COVID-19 infections.<br>Methodology: A case-control study design was employed. With this, a total of 261 COVID-19 deceased recordings were reviewed. The cases of the study were recordings of patients deceased within three days of intensive care unit admission whereas, the rest 187 were recordings of patients who died after three days of admission. Data were collected using an extraction checklist, entered into Epi data version 4.4.2.2, and analyzed by SPSS version 25. After the description, binary logistic regression was run to conduct bivariate and multivariable analyses. Finally, statistical significance was declared at p-value <0.05, and an adjusted odds ratio with a 95% confidence interval was used to report the strength of association.<br>Result: The analysis was performed on 261 (87 cases and 174 controls) with a response rate of 98.9%. About 62.5% of the participants were aged above 65 years and two-thirds were males. The presence of cardiovascular disease (AOR= 4.79, with 95%CI: 1.73, 13.27) and bronchial-asthma (AOR = 6.57; 95% CI: 1.39, 31.13) were found to have a statistically significant association with early mortality. The existence of complications from COVID-19 (AOR = 0.22; 95% CI: 0.07, 0.74) and previous history of COVID-19 infection (AOR=0.17, 95% CI: 0.04, 0.69) were associated with decreased risk of early mortality.<br>Conclusions: Having cardiovascular diseases and bronchial asthma was associated with an increased risk of early mortality. Conversely, the presence of intensive care unit complications and previous history of COVID-19 infection were associated with decreased risk of early mortality.
**Order of Authors:**	Taye Ashine Mezgebu, MSc
	Migbar Mekonnen Sibhat, Masters degree
	Melsew Getnet, PhD fellow
	Kassie Tiruneh Gebeyehu, Masters degree
	Wuletaw Zewde Chane, Sub specialist
	Edmialem Mesfin Getahun, Masters degree
	Asaminew Sane Habtamu, Masters degree
	Hailu Beyene Asmare, Masters degree
	Melke Mengistie Ambaw, Masters degree
**Additional Information:**	
**Question**	**Response**

Powered by Editorial Manager® and ProduXion Manager® from Aries Systems Corporation

**Financial Disclosure**<br><br>Enter a financial disclosure statement that describes the sources of funding for the work included in this submission. Review the submission guidelines for detailed requirements. View published research articles from *PLOS ONE* for specific examples.<br><br>This statement is required for submission and **will appear in the published article** if the submission is accepted. Please make sure it is accurate.<br><br>**Unfunded studies**<br>Enter: *The author(s) received no specific funding for this work.*<br><br>**Funded studies**<br>Enter a statement with the following details:<br>• Initials of the authors who received each award<br>• Grant numbers awarded to each author<br>• The full name of each funder<br>• URL of each funder website<br>• Did the sponsors or funders play any role in the study design, data collection and analysis, decision to publish, or preparation of the manuscript?<br>• **NO** - Include this sentence at the end of your statement: *The funders had no role in study design, data collection and analysis, decision to publish, or preparation of the manuscript.*<br>• **YES** - Specify the role(s) played.<br><br>* typeset	The authors received no specific funding for this work.
**Competing Interests**<br><br>Use the instructions below to enter a competing interest statement for this submission. On behalf of all authors, disclose any competing interests that could be perceived to bias this work—acknowledging all financial support and any other relevant financial or non-financial competing interests.<br><br>This statement is required for submission	The authors have declared that no competing interests exist.

Powered by Editorial Manager® and ProduXion Manager® from Aries Systems Corporation

and **will appear in the published article** if the submission is accepted. Please make sure it is accurate and that any funding sources listed in your Funding Information later in the submission form are also declared in your Financial Disclosure statement.<br><br>View published research articles from *PLOS ONE* for specific examples.<br><br>**NO authors have competing interests**<br><br>Enter: *The authors have declared that no competing interests exist.*<br><br>**Authors with competing interests**<br><br>Enter competing interest details beginning with this statement:<br><br>*I have read the journal's policy and the authors of this manuscript have the following competing interests: [insert competing interests here]*<br><br>* typeset	
**Ethics Statement**<br><br>Enter an ethics statement for this submission. This statement is required if the study involved:<br><br>• Human participants<br>• Human specimens or tissue<br>• Vertebrate animals or cephalopods<br>• Vertebrate embryos or tissues<br>• Field research<br><br>Write "N/A" if the submission does not require an ethics statement.<br><br>General guidance is provided below. Consult the submission guidelines for detailed instructions. **Make sure that all information entered here is included in the Methods section of the manuscript.**	Ethical clearance was obtained from Saint Paul's Hospital Medical College institutional review board (IRB). The IRB waived the study to be conducted by chart review. Since patient consent was not applicable due to the retrospective nature of the study, permission for data collection was obtained from the clinical director. The responsible bodies at each intensive care unit and liaison office were informed about the purpose of the study. The study was conducted per the declaration of Helsinki. Confidentiality and anonymity were assured by data coding and aggregate reporting, where no individual patient identifiers were included.

Powered by Editorial Manager® and ProduXion Manager® from Aries Systems Corporation

**Format for specific study types**<br><br>**Human Subject Research (involving human participants and/or tissue)**<br>• Give the name of the institutional review board or ethics committee that approved the study<br>• Include the approval number and/or a statement indicating approval of this research<br>• Indicate the form of consent obtained (written/oral) or the reason that consent was not obtained (e.g. the data were analyzed anonymously)<br><br>**Animal Research (involving vertebrate animals, embryos or tissues)**<br>• Provide the name of the Institutional Animal Care and Use Committee (IACUC) or other relevant ethics board that reviewed the study protocol, and indicate whether they approved this research or granted a formal waiver of ethical approval<br>• Include an approval number if one was obtained<br>• If the study involved *non-human primates*, add *additional details* about animal welfare and steps taken to ameliorate suffering<br>• If anesthesia, euthanasia, or any kind of animal sacrifice is part of the study, include briefly which substances and/or methods were applied<br><br>**Field Research**<br><br>Include the following details if this study involves the collection of plant, animal, or other materials from a natural setting:<br>• Field permit number<br>• Name of the institution or relevant body that granted permission	
**Data Availability**<br><br>Authors are required to make all data underlying the findings described fully available, without restriction, and from the time of publication. PLOS allows rare exceptions to address legal and ethical concerns. See the PLOS Data Policy and FAQ for detailed information.	Yes - all data are fully available without restriction

Powered by Editorial Manager® and ProduXion Manager® from Aries Systems Corporation

A Data Availability Statement describing where the data can be found is required at submission. Your answers to this question constitute the Data Availability Statement and **will be published in the article**, if accepted.<br><br>**Important:** Stating 'data available on request from the author' is not sufficient. If your data are only available upon request, select 'No' for the first question and explain your exceptional situation in the text box.<br><br>Do the authors confirm that all data underlying the findings described in their manuscript are fully available without restriction?	
**Describe where the data may be found in full sentences. If you are copying our sample text, replace any instances of XXX with the appropriate details.**<br><br>• If the data are **held or will be held in a public repository**, include URLs, accession numbers or DOIs. If this information will only be available after acceptance, indicate this by ticking the box below. For example: *All XXX files are available from the XXX database (accession number(s) XXX, XXX.*).<br>• If the data are all contained **within the manuscript and/or Supporting Information files**, enter the following: *All relevant data are within the manuscript and its Supporting Information files.*<br>• If neither of these applies but you are able to provide **details of access elsewhere**, with or without limitations, please do so. For example:<br><br>*Data cannot be shared publicly because of [XXX]. Data are available from the XXX Institutional Data Access / Ethics Committee (contact via XXX) for researchers who meet the criteria for access to confidential data.*<br><br>*The data underlying the results presented in the study are available from (include the name of the third party*	All relevant data are within the manuscript and its Supporting Information files are available upon reasonable request.

and contact information or URL).<br>• This text is appropriate if the data are owned by a third party and authors do not have permission to share the data.<br><br>* typeset	
Additional data availability information:	Tick here if your circumstances are not covered by the questions above and you need the journal’s help to make your data available.

Manuscript

Click here to access/download;Manuscript;Early mortality manuscript For PLOS ONE.docx

Risk Factors of early mortality among COVID-19 deceased patients in Addis Ababa COVID-19 Care Centers, Ethiopia

Taye Ashine Mezgebu[1*], Migbar Mekonnen Sibhat[2], Melsew Tsegaw Getnet[3], Kasie Tiruneh Gebeyehu[2], Wuletaw Zewde Chane[3], Edmialem Mesfin Getahun[3], Asaminew Sane Habtamu[4], Hailu Beyene Asmare[5,] Melke Mengistie Ambaw[6]

[1]Schools of Nursing, College of Health Science and Medicine, Wachemo University, Hosanna, Ethiopia

[2] College of Health Science and Medicine, Dilla University, Dilla, Ethiopia

[3]Mellinium COVID-19 Care Center, Addis Ababa, Ethiopia

[4]School of Nursing, College of Health Science and Medicine, Jimma University, Jimma, Ethiopia

[5]School of Nursing, College of Health Science and Medicine, Wolaita Sodo University, Sodo, Ethiopia

[6]Department of Nursing, Arba Minch College of sciences, Arba Minch, Ethiopia

*Corresponding author: Taye Ashine Mezgebu

E-mail: tayemezgebu26@gmail.com

Affiliation: Wachemo University, Hosanna, Ethiopia

Keywords: Intensive care unit, early mortality, COVID-19, risk factors, Ethiopia

Word count: 3121

Reference count: 35

Abstract

Background: Severe acute respiratory syndrome coronavirus-2 is a global health care problem with high mortality. Despite early mortality seeming alarming, data regarding factors that lead to increased early mortality of COVID 19 patients is not well-documented yet. The objective of this study was to identify the risk factors of early mortality in patients with confirmed COVID- 19 infections.

Methodology: A case-control study design was employed. With this, a total of 261 COVID-19 deceased recordings were reviewed. The cases of the study were recordings of patients deceased within three days of intensive care unit admission whereas, the rest 187 were recordings of patients who died after three days of admission. Data were collected using an extraction checklist, entered into Epi data version 4.4.2.2, and analyzed by SPSS version 25. After the description, binary logistic regression was run to conduct bivariate and multivariable analyses. Finally, statistical significance was declared at p-value <0.05, and an adjusted odds ratio with a 95% confidence interval was used to report the strength of association.

Result: The analysis was performed on 261 (87 cases and 174 controls) with a response rate of 98.9%. About 62.5% of the participants were aged above 65 years and two-thirds were males. The presence of cardiovascular disease (AOR= 4.79, with 95%CI: 1.73, 13.27) and bronchial-asthma (AOR = 6.57; 95% CI: 1.39, 31.13) were found to have a statistically significant association with early mortality. The existence of complications from COVID-19 (AOR = 0.22; 95% CI: 0.07, 0.74) and previous history of COVID-19 infection (AOR=0.17, 95% CI: 0.04, 0.69) were associated with decreased risk of early mortality.

Conclusions: Having cardiovascular diseases and bronchial asthma was associated with an increased risk of early mortality. Conversely, the presence of intensive care unit complications and previous history of COVID-19 infection were associated with decreased risk of early mortality.

Keywords: Intensive care unit, early mortality, COVID-19, risk factors, Ethiopia

Introduction

The emerged coronavirus disease-2019 (COVID-19), caused by the severe acute respiratory syndrome coronavirus-2 (SARS-CoV-2), has quickly spread worldwide with varied clinical characteristics (1). Until 20th February 2022, the COVID-19 pandemic has affected about 422 million cases and about 5.8 million deaths globally, covering more than 220 countries worldwide (2). However, Africa is forecasted to be the most defenseless continent where COVID-19 spreading will have a massive impact on the health system (3). In the same period, Ethiopia is burdened with 469,047 cases and 7,477 deaths. The clinical presentation and the mortality rate are widely varied across the countries. In countries like Ethiopia, with a diverse population, the clinical profile of COVID-19 is different even among different regions.

Patients with coronavirus disease 2019 (COVID-19) are perceived to have an increased risk of mortality in the early days of ICU admission before the causes of death are recognized. Different epidemiological studies had evidenced that the mortality rate was higher among ICU admitted patients than non-ICU patients (4-6). In the recent studies of COVID-19 patients from Wuhan, intensive care unit mortality rates ranged from 52% to 62% (7-10).

A study indicated that patients admitted early in the epidemic of COVID-19 had a higher mortality rate than patients admitted later (11). Some studies among non-COVID-19 patients demonstrated that the risk factors of early mortality are different from determinants of late mortality (12, 13). Shreds of evidence from plenty of studies showed that the risk factors regarding early mortality of COVID-19 are varied and may include male, older age, comorbidity, smoking, lymphocyte count, and D-dimer (13, 14). It is also hypothesized that delay in ICU admission could be one of the factors for early mortality after ICU admission (15). One-third of all patient deaths occur within three days of ICU admission (13).

Recognizing factors that can facilitate early mortality among patients admitted to the ICU settings is clinically significant. Identification of these factors aids in designing more effective ICU therapeutic strategies that aid to stabilize the patients' clinical condition, such as rescue therapy with advanced medical equipment and early human resources mobilization. Despite the clinical impact of early mortality is undisputable, studies assessing factors related to the early death of COVID-19 patients after ICU admission are scanty throughout the globe and in Ethiopia. Therefore, this study aims to identify risk factors for early mortality after ICU admission in patients with COVID-19.

Methods and Materials

A case-control study was carried out in Addis Ababa COVID-19 care centers, Ethiopia by reviewing charts of patients admitted from June 2020 to October 2021. There are three large COVID-19 care centers in Addis Ababa. Of those, Millennium COVID-19 care center is the largest center, with a capability of 1000 beds, including 40 ICU beds in three separated ICU setups. The source population was all patients who deceased due to COVID-19 in the centers, whereas all COVID-19 confirmed patients who deceased in ICU from June 2020 to October 2021 were the study population. The cases of the study (early mortality) were recordings of patients deceased within three days of ICU admission whereas; the controls (late mortality) were recordings of patients who deceased after three days of admission. All recordings of COVID-19 patients who were deceased in the ICU from June 2020 to October 2021 with complete records were included.

Sample size determination

The sample size was determined using Epi-Info version 7.2 by considering diabetes mellitus (DM) as a determinant variable from a previous study (13) which yields the maximum sample size. The parameters used include a 95% confidence level, 80% study power, an odds ratio of 2.32, a 16.87% proportion of DM among controls, and a 1:2 case to control ratio. Finally, the total sample size was 264 (88 cases and 176 controls). Then, cases were selected intentionally by the researcher, and two respective controls next to the selected cases were selected randomly using the systematic random sampling technique.

Data collection tools and procedures

Initially, the patient registries were evaluated to extract the total number of cases and to confine patients who died early and those who died late in the ICU. Then, the charts were arranged based on their medical record number (MRN) and the date of ICU admission. Afterward, the data were collected using a pretested and structured data extraction checklist which is designed from different literature (1, 13, 15-21). Then, the charts were arranged based on their medical record number (MRN) and the date of ICU admission. Afterward, the data were collected using a pretested and structured data extraction checklist which was newly designed with related variables. The checklist comprised socio-demographic characteristics, comorbidities, disease severity, baseline laboratory findings, and COVID-19 management-related variables. Six data collectors (MSc emergency and critical care nurses)

and two supervisors were recruited and trained for one about the requirements of data collection.

Analysis and interpretation of data

Data were cleaned, coded, and entered using Epi data version 4.4.2.2, and exported to SPSS version 25 for analysis. Cross-tabulation was done to determine the level of missing values and for data cleaning. Explorative analysis was conducted as well for continuous variables to determine the data distribution. Binary logistic regression was used to analyze the data. In the bivariate analysis, variables with a p-value of <0.2 were candidates for the final model for confounder adjustment. Multi-collinearity was checked using the variance inflation factor, and model fitness was checked using Hosmer and Lemeshow test (p=0.361). In the multivariate analysis, an adjusted odds ratio with 95% confidence intervals was computed to identify the presence and strength of associations. Statistical significance was stated at a p-value of <0.05.

Ethical considerations

Ethical clearance was obtained from Saint Paul's Hospital Medical College institutional review board (IRB). The IRB waived the study to be conducted by chart review. Since patient consent was not applicable due to the retrospective nature of the study, permission for data collection was obtained from the clinical director. The responsible bodies at each intensive care unit and liaison office were informed about the purpose of the study. The study was conducted per the declaration of Helsinki. Confidentiality and anonymity were assured by data coding and aggregate reporting, where no individual patient identifiers were included.

Results

This case-control study was conducted on 261 patients who died due to COVID-19 in the ICU (87 cases and 174 controls) with a response rate of 98.9%.

Socio-demographic characteristics and comorbidities

The study finding showed that 62.5% of the study participants aged above 65 years. Of those, 28.8% have died within the first 72 hours of ICU admission. The mean age of patients was 63.6years (SD=15.7) among the cases and 68.6 years (SD=13.7) among controls. On the other hand, about two-thirds (65.2%) of the patients were males, of which 32.4% passed away within three days of ICU admission (Table 1).

Table-1 Socio-demographic characteristics between early mortality and late mortality among COVID-19 patients at Addis Ababa COVID-19 care centers, Ethiopia, 2022 (n=261)

Variables	Category	Mortality		
		Early (%)	Late (%)	Total (%)
Age	<65 years	40 (40.8)	58 (59.2)	98 (37.5)
	≥65 years	47 (28.8)	116 (71.2)	163 (62.5)
Sex	Male	55 (32.4)	115 (67.6)	170 (65.2)
	Female	32 (35.2)	59 (64.8)	91 (34.8)

Description of comorbidities and other clinical characteristics

According to the study results, the proportion of severe disease was higher (83.3%) among those who died late after ICU admission compared to patients who died early. On the contrary, a higher proportion of patients admitted with moderate disease (25.3%) died early after ICU admission than those who died after three days (12%). The study finding also revealed that 44.3% of the controls had a history of previous COVID-19 infection, whereas only 8% of the cases had a history of COVID-19 infection. Regarding the preexisting medical illnesses, 183 (70.1%) of the patients had comorbidities, of which 83% had two or more comorbidities. The proportion of early mortality was much higher among patients with comorbid illnesses compared to those without comorbidities. The frequently documented chronic conditions were hypertension (35.6%), DM (35.2%), cardiovascular diseases (23.8%), and bronchial asthma (10.3%). Moreover, the proportion of patients who experienced COVID-19 complications was higher among the controls (77%) than in the cases (63%) (Table 2).

Table-2 Comorbidities and other clinical related characteristics between early mortality and late mortality among COVID-19 patients at Addis Ababa COVID-19 care centers, Ethiopia, 2022 (n=261)

Variables	Category	Early mortality (%)	Late mortality (%)	Total (%)
Smoking history	Yes	65 (41.9)	90 (58.1)	155 (59.4)
	No	22 (20.8)	84 (79.2)	106 (40.6)
Triage severity category	Mild	2 (20.0)	8 (80.0)	10 (3.8)
	Moderate	22 (51.2)	21 (48.8)	43 (16.5)
	Severe	63 (30.3)	145 (69.7)	208 (79.7)

History of previous COVID-19 infection	Yes	7 (8.3)	77 (91.7)	84 (32.2)
	No	80 (45.2)	97 (54.8)	177 (67.8)
Number of comorbidities	None	11 (14.1)	67(85.9)	78 (29.9)
	One	10 (30.3)	23 (69.7)	33 (12.6)
	≥ two	66 (44.0)	84 (56.0)	150 (57.5)
Diabetics Mellitus	Yes	42 (45.7)	50 (54.3)	92 (35.2)
	No	45 (26.6)	124 (73.4)	169 (64.8)
Hypertension	Yes	39 (41.9)	54 (58.1)	93 (35.6)
	No	48 (28.6)	120 (71.4)	168 (64.4)
Cardiovascular disease	Yes	38 (61.3)	24 (38.7)	62 (23.8)
	No	49 (24.6)	150 (75.4)	199 (76.2)
Bronchial asthma	Yes	14 (51.9)	13 (48.1)	27 (10.3)
	No	73 (31.2)	161 (68.8)	234 (89.7)
COPD	Yes	8 (100)	0	8 (3.1)
	No	79 (31.2)	174 (68.8)	253 (96.9)
Malignancy	Yes	11 (37.9)	18 (62.1)	29 (11.1)
	No	76 (32.8)	156 (67.2)	232 (88.9)
Renal disease	Yes	5(50.0)	5 (50.0)	10 (3.8)
	No	82 (32.7)	169 (67.3)	251 (96.2)
ICU admission delay	Yes	39 (56.5)	30 (43.5)	69 (26.4)
	No	48 (25.0)	144 (75.0)	192 (73.6)
Complication of COVID-19	Yes	55 (29.1)	134 (70.9)	189 (72.4)
	No	32 (44.4)	40 (56.2)	72 (27.6)

Abbreviation: ICU, Intensive Care Unit; COPD, chronic obstructive pulmonary disease; COVID-19-coronavirus 2019

Description of baseline vital signs and laboratory findings

Concerning baseline vital signs, about 88.1%, 50.6%, and 73.2% of the patients were tachypneic, tachycardia, and hypoxic at admission. Among patients who died within 72 hours of ICU admission, 56.3%, 55.2%, and 31% had leukocytosis, anemia, and thrombocytopenia. The average white blood cell count at admission was 15.71×10^3c/µL (SD=16.70) among the cases and 14.82×10^3c/µL (SD=11.30) among the controls. Furthermore, the mean serum albumin was 2.55g/dl (SD=1.40) among patients who died within the first 72 hours of ICU admission and 2.6g/dl (SD=0.92) among patients who died 3 days later after admission.

Almost two-thirds of the cases had random blood sugar levels of >200mg/dl, whereas it was noted among just one-third of the controls (Table 3).

Table-3 Baseline vital sign parameters and laboratory finding between early mortality and late mortality among COVID-19 patients at Addis Ababa COVID care centers, Ethiopia, 2022 (n=261)

Variables	Category	Cases (%)	Controls (%)	Total (%)
Respiratory rate (bpm)	<24	7 (22.6)	24 (77.4)	31 (11.9)
	≥24	80 (34.8)	150 (65.2)	230 (88.1)
Systolic blood pressure (mmHg)	<90	6 (16.2)	31 (83.8)	37 (14.2)
	≥90	81 (36.2)	143 (63.8)	224 (85.8)
Pulse rate beat per minute	<100	38 (29.5)	91 (70.5)	129 (49.4)
	≥100	49 (37.4)	83 (62.9)	132 (50.6)
Oxygen saturation (%)	<90	68 (35.6)	123 (64.4)	191 (73.2)
	≥90	19 (27.1)	51 (72.9)	70 (26.8)
Temperature (℃)	<37.5	85 (35.9)	152 (64.1)	237 (90.8)
	≥37.5	2 (8.3)	22 (91.7)	24 (9.2)
White blood cell count (cells/uL)	<11,000	38 (35.5)	69 (64.5)	107 (41.0)
	≥11,000	49 (31.8)	105 (68.2)	154 (59.0)
Hemoglobin (milligram/dl)	<12	48 (49.0)	50 (51.0)	98 (37.5)
	≥ 12	39 (23.9)	124 (76.1)	163 (62.5)
Platelet count (cells/uL)	<150,000	27 (42.9)	36 (57.1)	63 (24.1)
	≥150,000	60 (30.3)	138 (69.7)	198 (75.9)
Creatinine, (milligram/dL)	≤1.2	50 (25.6)	145 (74.4)	195 (74.7)
	> 1.2	37 (56.1)	29 (43.9)	66 (25.3)
Blood urea nitrogen (mg/dl)	<45	35 (26.9)	95 (73.1)	130 (49.8)
	≥45	52 (39.7)	79 (60.3)	131 (50.2)
Alanine aminotransferase (U/L)	<40	45 (24.2)	141 (75.8)	186 (71.3)
	≥ 40	42 (56.0)	33 (44.0)	75 (28.7)
Serum potassium (mmol/L)	<3.5	0	8 (100)	8 (3.1)
	3.5-5.0	60 (33.7)	118 (66.3)	178 (68.2)
	> 5	27 (36.0)	48 (64.0)	75 (28.7)
Serum sodium (mmol/L)	<135	22 (41.5)	31 (58.5)	53 (20.3)
	135-146	65 (31.3)	143 (68.8)	208 (79.7)
Albumin (gram/dl)	<3.4	68 (33.2)	137 (66.8)	205 (78.5)
	≥3.4	19 (33.9)	37 (66.1)	56 (21.5)
Random blood sugar (milligram /dl)	<200	31 (22.6%)	106 (77.4)	137 (525)
	≥200	56 (45.2)	68 (54.8)	124 (47.5)
Chest radiological finding	Unilateral infiltration	18 (19.8)	73 (80.2)	91 (34.9)
	Bilateral infiltration	69 (40.6)	101 (59.4)	170 (65.1)

Abbreviation: ICU, intensive care unit; bpm, breath per minute; ℃, degree celsius; dl, deciliter; mEq/L, mill equivalent per liter; mg/dl, mill gram per deciliter.

COVID-19 management-related factors

Among the study participants, 62.1% of patients were intubated. Of those, 31% died within three days of ICU admission. The proportion of patients who received vasopressor therapy was higher among those who died late after ICU admission (59.8%) than those who died early (44.8%). Prone position was assumed by 40.2% of the cases and 74.1% of the controls. In addition, 95.1%, 95.4%, 85.4%, and 23.4% of the patients took systemic antibiotics, corticosteroid therapy, thromboprophylaxis, and antivirals respectively (Table 4).

Table-4 Treatment at ICU admission among early and late mortality of ICU admitted COVID-19 at Millennium COVID Care Center, Addis Ababa, Ethiopia 2022 (n=261).

Variables	**Category**	**Cases (%)**	**Controls (%)**	**Total (%)**
Mode of ventilation	None invasive	37 (37.4)	62 (62.6)	99 (37.9)
	Intubated	50 (30.9)	112 (69.1)	162 (62.1)
Broad spectrum Antibiotics	Yes	74 (29.8)	174 (70.2)	248 (95.1)
	No	13 (100)	0 (0%)	13 (4.9)
Vasopressors,	Yes	39 (27.3)	104 (72.7)	143 (54.8)
	No	48 (40.7)	70 (70.1)	118(45.2)
Systemic steroid	Yes	81 (32.5)	168 (67.5)	249 (95.4)
	No	6 (50.0)	6 (50.0)	12 (4.6)
Thromboprophylaxis	Yes	69 (30.9)	154 (69.1)	223 (85.4)
	No	18 (47.4)	20 (52.6)	38 (14.6)
Antihypertensive	Yes	19 (32.2)	40 (67.8)	59 (22.6)
	No	68 (33.7)	134 (66.3)	202 (77.4)
Antiviral	Yes	11 (18.0)	50 (82.0)	61 (23.4)
	No	76 (38.0)	124 (62.0)	200 (76.6)
Prone position	Yes	35 (21.3)	129 (80.0)	164 (62.8)
	No	52 (53.6)	45 (46.4)	97 (37.2)
Blood transfusion	Yes	0	29 (100)	29 (11.1)
	No	87 (37.5)	145 (62.5)	232 (88.8)
Hemodialysis	Yes	11 (20.0)	44 (80.0)	55 (21.1)
	No	76 (36.9)	130 (63.1)	206 (78.9)

Risk factors of early mortality

After data description, bivariate and multivariable analyses were performed using the binary logistic regression model. Variables with a p-value of <0.25 in the bivariate analysis were candidates for confounder adjustment in the final model. After checking the model fitness, the multivariable analysis was run. In the multivariate analysis, COVID19 complications, cardiovascular disease (CVD), bronchial asthma, and previous history of COVID-19 infection showed statistically significant association with early mortality of COVID-19 patients at a 95% confidence level.

Patients having CVD were 4.79 times (AOR= 4.79; 95%CI: 1.73, 13.27) more lik[illegible]o die within the first 72 hours of ICU admission compared to those who had no CVD. Likewise, patients with bronchial asthma had 6.57 times increased chance of early mortality compared to their counterparts (AOR = 6.57; 95% CI: 1.39, 31.13). On the contrary, the odds of early mortality among patients who had a history of previous COVID-19 infection were 83% decreased compared to their counterparts (AOR=0.17; 95% CI: 0.04, 0.69). The other variable that showed significant association with early mortality was the presence of COVID-19 complications. Patients who experienced COVID-19 complications were 78% times less likely to die within the first three days than after then (Table 5).

Table-5 Bivariate and multivariate analysis of risk factors associated with early mortality after ICU admission among COVID-19 patients at Addis Ababa COVID 19 Care Centers, Ethiopia, 2022 (n= 261)

**Risk factors**	**Category**	**Mortality**		**COR (95%CI)**	**AOR (95%CI)**	**Sig.**
		**Late**	**Early**			
Age	<65 years	58	40	1	1	1
	≥65 years	116	47	0.59 (0.35, 0.99)	0.51 (0.19, 0.1.32)	0.165
Triage score	Not severe	24	29	1	1	1
	Severe	63	145	0.53 (0.28- 0.97)	0.48 (0. 18, 1.27)	0.138
Complication of COVID-19	Yes	134	55	1.95 (1.11, 3.42)	0.22 (0.08, 0.59)	**0.003***
	No	40	32	1	1	1
Prone position	Yes	129	35	0.24 (0.14, 0.41)	0.56 (0.15, 2.08)	0.384
	No	45	52	1	1	
History of previous COVID-19 infection	Yes	77	7	9.07 (0.3.96, 20.77)	0.17 (0.04, 0.69)	**0.013***
	No	97	80	1	1	1
Diabetes Mellitus	Yes	29	81	2.32 (01.36, 3.95)	0.96 (0.34, 2.68)	0.934
	No	28	32	1	1	1
Hypertension	Yes	25	34	1.82 (.94,3.51)	1.65 (0.53, 5.13)	0.388
	No	32	79	1	1	
Cardiovascular disease	Yes	24	14	4.85 (2.65, 8.87)	4.79 (1.73, 13.27)	**0.003***
	No	33	99	1	1	

Bronchial asthma,	Yes	13	14	2.38 (1.06, 7.53)	6.569 (1.39, 31.13)	**0.018***
	No	161	73	1	1	1
ICU admission delay	Yes	30	39	0.26 (0.14, 0.46)	1.41 (0.53, 3.71)	0.491
	No	144	48	1	1	
Vasopressor	Yes	104	39	0.55 (0.33, 0.92)	0.85 (0.33, 2.18)	0.728
	No	70	48	1	1	1
Pulse rate (beat per	<100	91	38	1	1	1
minute)	≥100	83	49	1.41 (0.84, 2.37)	2.89 (0.92, 9.12)	0.070
Peripheral oxygen	<90	123	68	1.48 (0.811, 2.716)	0.52 (0.12, 2.19)	0.372
saturation (%)	≥90	51	19	1	1	1
Creatinine (mg/dl)	<1.2	145	50	1	1	1
	≥ 1.2	29	37	3.70 (2.07, 6.63)	1.91 (0.48, 7.53)	0.358
Platelet count	<150,000	36	27	1.73 (0.95, 3.09)	1.52 (0.56, 4.10)	0.414
(cells/µl)	≥150,000	138	60	1	1	1
Alanine amino-	<40	141	45	1	1	1
transferase (U/L)	≥ 40	33	42	2.29 (1.23, 4.28)	1.30 (0.44, 3.86)	0.632
Hemoglobin (g/dl)	<12	50	48	3.05 (1.79, 5.21)	1.12 (0.38, 3.29)	0.840
	≥ 12	124	39	1	1	1
Serum sodium	>135	31	22	1.56 (0.84, 2.90)	1.72 (0.51, 5.80)	0.385
(mmol/L)	135-145	143	65	1	1	1
Random blood sugar	<200	106	31	1	1	1
(mg/dl)	≥200	68	56	0.37 (0 .19, 0.72)	2.09 (0.59, 7.39)	0.253

Note * significantly associated with early mortality

Abbreviation: COR, crude odds ratio; AOR, adjusted odds ratio; CI, confidence interval; COVID-19, coronavirus disease 2019; ICU, intensive care unit

Discussion

Existing evidence showed that several COVID-19 patients died within the first three days of admission (22, 23). Identifying the factors for this increased death in the early stages of ICU admission would enable us to set evidence-based interventions and reduce this problem. However, to the best of our knowledge, no study specifically investigated the factors contributed to the increased mortality in the early stages of hospital admission compared to those who died lately. Thus, this case-control study assessed the risk factors of early mortality among COVID-19 patients at the intensive care unit of Addis Ababa COVID-19 care centers. After adjusting for potential confounders, four variables were found to be independent risk factors of early mortality among COVID-19 ICU patients.

Patients having CVD were 4.79 times (AOR= 4.79; 95%CI: 1.73, 13.27) more like to die within the first 72 hours of ICU admission compared to those who had no CVD. This finding is consistent with the study conducted in Pakistan (16) and Wuhan, China (8, 24). However, these studies investigated the factors associated with mortality of COVID-19 patients as a

whole and were not specific to early mortality. The possible justification could be the effect of COVID-19 on the cardiovascular system. Severe COVID-19 disease is associated with hemodynamic instabilities, severe ARDS (requiring high PEEP levels of ventilation), decreased cardiac output, hypervolemia, sepsis, and those who received nephrotoxic drugs (25). These effects may, in turn, worsen the preexisting cardiovascular disease leading to more severe illness, cardiac arrest, and higher mortality (23).

Likewise, patients with bronchial asthma had 6.57 times increased chance of early mortality compared to their counterparts (AOR=6.57; 95% CI: 1.39, 31.13). This finding was conformable with previous studies in the United Kingdom (26) and Korea (27) that reported a higher risk of COVID-19 severity and mortality among asthmatic patients. The reason for this might be that COVID-19 infection trigger acute exacerbation of bronchial asthma that leads to oxygen hunger. Hence, hypoxia can result in respiratory failure ending up in premature death. However, the findings of studies conducted in France (28) and New York City (29) contradict the current study result. This discrepancy might be due to the difference in the study subjects. Unlike in our study, previous studies reported factors for overall COVID-19 mortality (merging both late and early mortality). It needs additional study to clear the role of asthma on COVID-19 patients' mortality.

On the contrary, the odds of early mortality among patients who had a history of previous COVID-19 infection were 83% decreased compared to their counterparts (AOR=0.17; 95% CI: 0.04, 0.69). This finding is supported by studies done in the USA (30), Australia (31), and the UK (32) that showed a low reinfection rate, morbidity, and mortality among those who had a history of previous COVID-19 infection. This might be due to the development of antibodies and strengthened immune systems after being infected by COVID-19, which declines the probability of severe disease (33, 34). Even though we could not find former reports regarding the association between early mortality and previous history of COVID-19 infection, this finding magnifies the impact of COVID-19 vaccination to boost immunity and patient outcome.

The other variable that showed significant association with early mortality was the presence of COVID-19 complications. Patients who experienced COVID-19 complications had a 78% reduced mortality risk within the first 72 hours than after three days of ICU admission. The rationale could be that most fatal ICU complications such as septic shock, acute kidney injury, hospital-acquired infections, and ventilator-associated pneumonia occur late in the ICU (after 48-72 hours of admission) after intubation (35). Furthermore, even patients who

developed ICU complications in the early days may die after three days and counted as late mortality. Hence, this high record of complication-associated deaths in the late mortality group might conceal the effect of complications on the early mortality group despite its impact on poor patient outcomes, in general, being undisputable. To strengthen this rationale, deaths linked to COVID-19 complications were more prevalent among the late mortality group in this study.

While using and interpreting the findings of this study, the following issues should be taken into consideration. The main limitation of this study was the lack of consideration of the duration from symptom onset up to hospital admission. Irrespective of the possible factors for early mortality, patients who had stayed longer to seek medical attention might not have a similar length of hospital stay and in-hospital outcome compared to those who did not delay. Secondly, as the samples were recruited based on the outcome status, it could be quite difficult to evaluate the effect of some variables (e.g. ICU complications) that might affect the survival and timing of mortality. Besides, the general limitation of retrospective studies could be another point to be reminded of while applying the study findings.

Conclusion

Having cardiovascular diseases and bronchial asthma was associated with an increased risk of early mortality. Conversely, the presence of ICU complications and previous history of COVID-19 infection were found to have a protective effect on early mortality. Hence, it is essential to put specific strategies to manage and halt the identified factors to minimize the high burden of early mortality among COVID-19 patients.

Abbreviations: AOR, Adjusted Odd Ratio; ARDS, Acute Respiratory Distress Syndrome; CI, Confidence Interval; COPD, Chronic Obstructive Pulmonary Disease; COVID-19, coronavirus disease-2019; DM, Diabetics Mellitus; ICU, Intensive Care Unit; IRB, Institutional Review Board; MSc, Master of Science; MRN, Medical Record Number; PEEP, Positive End Expiratory Pressure; SD, Standard Deviation; SPSS, Statistical Package for the Social Sciences; the USA, United States of America

Acknowledgments: The center administrators, staff, data collectors, and supervisors were appreciated for providing the necessary preliminary information. The authors would also like to thank Saint Paul's Hospital Millennium Medical College to pursue the chance to conduct this study.

Funding: This research received no specific funding from any organization in the public, commercial, or not-for-profit sectors.

Authors' contributions: Initially, TMA comes with the study topic. TMA, AHS, KGT, and MSM were involved in the study design and tool preparation. TMA wrote the research proposal. MGT, MSM, & WCZ edited and revised the proposal. Then, TMA, HAB, KGT, MAM, EGM, AHS, and WCZ participated in the data collection and entry. TMA, AHA, MGT & MSM conducted the statistical analysis, and TMA drafted the manuscript. MSM, MGT, & AHS edited the manuscript and formatted it for publication. Afterward, all the authors read, critically revised, approved the manuscript, and agreed to be accountable for all aspects of this research.

Disclosure: The authors declared no conflicts of interest.

Data sharing statement: Extra data that support the findings of this study are accessible from the corresponding author upon reasonable request and can be shared upon legal request via tayemezgebu26@gmail.com

Consent for publication: Not applicable

References

1. Zhang X-B, Hu L, Ming Q, Wei X-J, Zhang Z-Y, Chen L-D, et al. Risk factors for mortality of coronavirus disease-2019 (COVID-19) patients in two centers of Hubei province, China: A retrospective analysis. PloS one. 2021;16(1):e0246030.
2. Organization WH. COVID-19 weekly epidemiological update, edition 80, 22 February 2022. 2022.
3. Lone SA, Ahmad A. COVID-19 pandemic–an African perspective. Emerging microbes & infections. 2020;9(1):1300-8.
4. Urra J, Cabrera C, Porras L, Ródenas I. Selective CD8 cell reduction by SARS-CoV-2 is associated with a worse prognosis and systemic inflammation in COVID-19 patients. Clinical immunology. 2020;217:108486.
5. Grasselli G, Greco M, Zanella A, Albano G, Antonelli M, Bellani G, et al. Risk factors associated with mortality among patients with COVID-19 in intensive care units in Lombardy, Italy. JAMA internal medicine. 2020;180(10):1345-55.
6. Ayed M, Borahmah AA, Yazdani A, Sultan A, Mossad A, Rawdhan H. Assessment of clinical characteristics and mortality-associated factors in COVID-19 critical cases in Kuwait. Medical Principles and Practice. 2021;30(2):185-92.

7. Wu C, Chen X, Cai Y, Zhou X, Xu S, Huang H, et al. Risk factors associated with acute respiratory distress syndrome and death in patients with coronavirus disease 2019 pneumonia in Wuhan, China. JAMA internal medicine. 2020;180(7):934-43.
8. Zhou F, Yu T, Du R, Fan G, Liu Y, Liu Z, et al. Clinical course and risk factors for mortality of adult inpatients with COVID-19 in Wuhan, China: a retrospective cohort study. The lancet. 2020;395(10229):1054-62.
9. Huang C, Wang Y, Li X, Ren L, Zhao J, Hu Y, et al. Clinical features of patients infected with 2019 novel coronavirus in Wuhan, China. The lancet. 2020;395(10223):497-506.
10. Yang X, Yu Y, Xu J, Shu H, Liu H, Wu Y, et al. Clinical course and outcomes of critically ill patients with SARS-CoV-2 pneumonia in Wuhan, China: a single-centered, retrospective, observational study. The Lancet Respiratory Medicine. 2020;8(5):475-81.
11. van Halem K, Bruyndonckx R, van der Hilst J, Cox J, Driesen P, Opsomer M, et al. Risk factors for mortality in hospitalized patients with COVID-19 at the start of the pandemic in Belgium: a retrospective cohort study. BMC infectious diseases. 2020;20(1):1-10.
12. Burgos J, Luján M, Larrosa MN, Pedro-Botet ML, Fontanals D, Quesada MD, et al. The problem of early mortality in pneumococcal pneumonia: a study of risk factors. European Respiratory Journal. 2015;46(2):561-4.
13. Daviaud F, Grimaldi D, Dechartres A, Charpentier J, Geri G, Marin N, et al. Timing and causes of death in septic shock. Annals of intensive care. 2015;5(1):1-9.
14. Pinato DJ, Lee AJ, Biello F, Seguí E, Carbó A, Bruna R, et al. Presenting features and early mortality from SARS-CoV-2 infection in cancer patients during the initial stage of the COVID-19 pandemic in Europe. Cancers. 2020;12(7):1841.
15. Driessen RG, Heijnen NF, Hulsewe RP, Holtkamp JW, Winkens B, van de Poll MC, et al. Early ICU-mortality in sepsis–causes, influencing factors and variability in clinical judgement: a retrospective cohort study. Infectious Diseases. 2021;53(1):61-8.
16. Ayaz A, Arshad A, Hajra Malik HA, Hussain E, Jamil B. Risk factors for intensive care unit admission and mortality in hospitalized COVID-19 patients. Acute and critical care. 2020;35(4):249.
17. Nishikimi M, Rasul R, Sison CP, Jafari D, Shoaib M, Shinozaki K, et al. Intubated COVID-19 predictive (ICOP) score for early mortality after intubation in patients with COVID-19. Scientific reports. 2021;11(1):1-11.

18. Morell-Garcia D, Ramos-Chavarino D, Bauça JM, Argente del Castillo P, Ballesteros-Vizoso MA, Garcia de Guadiana-Romualdo L, et al. Urine biomarkers for the prediction of mortality in COVID-19 hospitalized patients. Scientific Reports. 2021;11(1):1-13.

19. Gao J, Zhong L, Wu M, Ji J, Liu Z, Wang C, et al. Risk factors for mortality in critically ill patients with COVID-19: a multicenter retrospective case-control study. BMC Infect Dis. 2021 Jun 24;21(1):602. PubMed PMID: 34167463. Pubmed Central PMCID: PMC8223178. Epub 2021/06/26. eng.

20. Leoni MLG, Lombardelli L, Colombi D, Bignami EG, Pergolotti B, Repetti F, et al. Prediction of 28-day mortality in critically ill patients with COVID-19: Development and internal validation of a clinical prediction model. Plos one. 2021;16(7):e0254550.

21. Li J, Yang L, Zeng Q, Li Q, Yang Z, Han L, et al. Determinants of mortality of patients with COVID-19 in Wuhan, China: a case-control study. Annals of Palliative Medicine. 2021.

22. Yen Y-F, Chan S-Y, Chen C-C, Deng C-Y. Predictors for Early and Late Death in Adult Patients with COVID-19: A Cohort Study. International journal of environmental research and public health. 2022;19(6):3357.

23. Xie M, Sun W, Zhang Y, Wu C, Wang S, Xie Y, et al. Early versus Late Onset Cardiac Injury and Mortality in Hospitalized COVID-19 Patients in Wuhan. Frontiers in Cardiovascular Medicine. 2021;8:333.

24. Shi S, Qin M, Shen B, Cai Y, Liu T, Yang F, et al. Association of cardiac injury with mortality in hospitalized patients with COVID-19 in Wuhan, China. JAMA cardiology. 2020;5(7):802-10.

25. Gabarre P, Dumas G, Dupont T, Darmon M, Azoulay E, Zafrani L. Acute kidney injury in critically ill patients with COVID-19. Intensive care medicine. 2020 Jul;46(7):1339-48. PubMed PMID: 32533197. Pubmed Central PMCID: PMC7290076. Epub 2020/06/14. eng.

26. Choi HG, Wee JH, Kim SY, Kim JH, Kim HI, Park JY, et al. Association between asthma and clinical mortality/morbidity in COVID- 19 patients using clinical epidemiologic data from Korean Disease Control and Prevention. Allergy. 2021.

27. Yang JM, Koh HY, Moon SY, Yoo IK, Ha EK, You S, et al. Allergic disorders and susceptibility to and severity of COVID-19: a nationwide cohort study. Journal of Allergy and Clinical Immunology. 2020;146(4):790-8.

28. Beurnier A, Jutant E-M, Jevnikar M, Boucly A, Pichon J, Preda M, et al. Characteristics and outcomes of asthmatic patients with COVID-19 pneumonia who require hospitalisation. European Respiratory Journal. 2020;56(5).
29. Lieberman-Cribbin W, Rapp J, Alpert N, Tuminello S, Taioli E. The impact of asthma on mortality in patients with COVID-19. Chest. 2020;158(6):2290-1.
30. Kojima N, Shrestha N, Klausner JD. A systematic review of the protective effect of prior SARS-CoV-2 infection on repeat infection. Evaluation & the Health Professions. 2021;44(4):327-32.
31. Pilz S, Chakeri A, Ioannidis JP, Richter L, Theiler- Schwetz V, Trummer C, et al. SARS- CoV- 2 re- infection risk in Austria. European journal of clinical investigation. 2021;51(4):e13520.
32. Mensah AA, Lacy J, Stowe J, Seghezzo G, Sachdeva R, Simmons R, et al. Disease severity during SARS-COV-2 reinfection: a nationwide study. Journal of Infection. 2022.
33. Dagan N, Barda N, Kepten E, Miron O, Perchik S, Katz MA, et al. BNT162b2 mRNA Covid-19 vaccine in a nationwide mass vaccination setting. New England Journal of Medicine. 2021.
34. Hall VJ, Foulkes S, Charlett A, Atti A, Monk EJ, Simmons R, et al. SARS-CoV-2 infection rates of antibody-positive compared with antibody-negative health-care workers in England: a large, multicentre, prospective cohort study (SIREN). The Lancet. 2021;397(10283):1459-69.
35. Lagoe RJ, Johnson PE, Murphy MP. Inpatient hospital complications and lengths of stay: a short report. BMC research notes. 2011;4:135-. PubMed PMID: 21545741. eng.
