# Supplementary material for: Risk factors of early mortality among COVID-19 deceased patients in Addis Ababa COVID-19 care centers, Ethiopia
Source: PLoS One. 2022 Sep 27;17(9):e0275131. doi: 10.1371/journal.pone.0275131 (PMC9514640; doi:10.1371/journal.pone.0275131)
Supplement: Attachment — Submitted filename: Response to Reviewers EM.docx [file pone.0275131.s003.docx]

**Manuscript title:** Risk Factors of early mortality among COVID-19 deceased patients in Addis Ababa COVID-19 Care Centers, Ethiopia (ID PONE-D-22-10705)

**Response to Editor and Reviewers’ Comments**

Dear editor and reviewers of PLOS ONE journal,

We the authors of this manuscript would like to appreciate the editor and reviewers for your thoughtful feedback and comments. We are pleased with your immediate response as well. Here below are responses to the comments.

**Editorial comments**

1. **Comment #1:** “Please ensure that your manuscript meets PLOS ONE's style requirements, including those for file naming."

**Author’s response:** We have reviewed the journal styles and requirements and adjust the files accordingly.

2. **Comment #2:** “We note that you have indicated that data from this study are available upon request. PLOS only allows data to be available upon request if there are legal or ethical restrictions on sharing data publicly.”

**Author’s response:** We read the PLOS ONE data sharing policy. The data contain potentially identifying or sensitive patient information on human participants in a specified setting. Due to this reason, the data could not be shared publicly. However, if it is needed for the publication process, the concerned body (Either the Editor or the reviewers’) can request and access the minimal anonymized dataset from the corresponding author via [bayayibignabez@gmail.com](mailto:bayayibignabez@gmail.com). Accordingly, we specified contact information to access the data from the corresponding author in the manuscript file under “data availability statement” (Line 328-330).

3. **Comment #3: “**PLOS requires an ORCID iD for the corresponding author in Editorial Manager on papers submitted after December 6th, 2016. Please ensure that you have an ORCID iD and that it is validated in Editorial Manager.”

**Author’s response:** Thank you! We have linked an ORCiD ID of the corresponding author to the editorial manger site as recommended.

4. **Comment #3:** “Please review your reference list to ensure that it is complete and correct. If you have cited papers that have been retracted, please include the rationale for doing so in the manuscript text, or remove these references and replace them with relevant current references.”

**Author’s response:** The whole references listed were reviewed for completeness and appropriateness.

**Reviewer #1: Reflections**

1. **Comment:** “Were participants and/or researchers blind?”

**Author’s response:** Of course, we can say it was anonymous! The researchers did not directly contact the patients and patient families. The data collection was conducted via patient chart review retrospectively after permission granted from the hospital administrators on behalf of patients.

2. **Comment:** “On what type of significance is the focus in the presentation of results: statistical or clinical?”

**Author’s response:** To be direct forward, the primary focus in the presentation of findings was clinical significance whenever inconsistencies happen. However, we tried to match both statistical and clinical significances together if possible.

3. **Comment: “**Line 90: Delete despite, start with International evidence suggests that ....and find some references”

**Author’s response:** Thank you! We appreciate the suggestion and we have cited the references as recommended (Line 90). On the other hand, rephrasing the statement could completely alter the main idea of the sentence and prefer to keep as it is.

4. **Comment: “**When was the diagnosis made, that is important before ICU admission”

**Author’s response:** Since the study was on severely or critically ill patients, the diagnosis of those patients was made immediately before admission to the ICU. Because, once diagnosed they will be immediately admitted to the ICU. However, if the respected reviewer intends to ask the time of COVID-19 diagnosis, it may vary from patient to patient such that some patients might be directly diagnosed with severe/critical illness and some others might come with mild disease before ICU admission secondary to deterioration. But, in this study the follow-up will begin at the time of ICU admission.

5. **Comment:** Please explain, what you mean by intentionally, as you also mention randomly selecting the patients.

**Author’s response:** Since the study was case-control study, we have applied different sampling techniques for cases and controls. Accordingly, we have selected the cases (early mortality) intentionally. Nonetheless, controls (lately deceased) patients were selected randomly using systematic random sampling technique as already stated in the manuscript (Please refer to Line 119-121). This is because we may have several late mortality records to apply interval selection.

6. **Comment:** “Line 151: delete have”

**Author’s response:** We made the required change (See Line 162).

7. **Comment:** “Line 158: “what parameters were used as mild, moderate and severe disease?”

**Author’s response:** The parameters used to classify as mild, moderate, and severe/critical include clinical, radiographic, and laboratory findings based on WHO guideline for management of COVID-19 patients. We have provided the operational definition of this classification in the main manuscript file to make it clear (Please refer Line 105-113).

8. **Comment:** “Line 163: This sentence is a bit confusing, please rephrase, I assume you are trying to say whereas only 8% of patients with early mortality ......”

**Author’s response:** The statement was rephrased in clear terms (see Line 174 &175).

9. **Comment:** “Line 169: rephrase to than in the cases with early mortality”

**Author’s response:** Sentence rephrased as suggested (Line 181).

10. **Comment:** “Table 2: What parameters were looked with regards to complications of COVID-19.”

**Author’s response:** Thanks again! We have jotted-down the COVID-19 complications recorded and specified in the table footnote (Line 186 & 187).

11. **Comment:** “Line 181: Reword: cases to with early mortality”

**Author’s response:** Moderated as suggested (Line 195).

12. **Comment:** “Line 184: Reword cases to: early mortality case 208: typing error most likely: please change like to likely”

**Author’s response:** Modified as recommended (Line 198, 222).

13. **Comment:** “Line 216: What parameters did you look at as "COVID complications"? Please name them however leave it under one group as mentioned in the table.”

**Author’s response:** Parameters were listed-out in the narration as recommended (Please refer to 229 & 230).

14. **Comment:** “Sentence that starts in line 227: Is this statement completely true? Perhaps no such study has been conducted in Ethiopia would be more appropriate.”

**Author’s response:** Comment accepted and required changes were made (Line 244).

15. **Comment:** “Last sentence beginning in line 252: Rephrase, to additional studies are therefore required to completely elucidate the role of asthma in early mortality or leave it out completely as you have discussed why other findings are different from yours.”

**Author’s response:** Thank you in advance! We have accepted the comments and rephrased as suggested (Line 268 & 269).

16. **Comment: “**Line 270: delete counted and rather say were recorded or were grouped under the late mortality group based on the criteria of early vs late mortality.”

**Author’s response:** The required changes were made accordingly (286).

**Reviewer #2: Reflections**

1. **In the ABSTRACT part:**

- **Comment #1:** “Is this study a Case-Control? All patients have the disease, therefore there is no ‘Control’ group without the disease. Consider re-classifying the study type. This study is a retrospective review of patient files, the authors need to be very clear about this.”

**Author’s responses:** Yes, of course! The aim of the study was to identify the determinant factors of early mortality. Hence, the population comprised of COVID-19 patients who were deceased in the ICU. Accordingly, we have classified recordings of patients died in the first 72 hours after ICU admission as cases (early mortality) and deaths recorded after 72 hours of ICU admission were considered as controls (late mortality) **(Kindly refer to line 101-103).** This implies, early mortality were cases (with disease) and late mortality were controls (without disease).

- **Comment #2:** “What is the ‘response rate’ referring to? This normally applies to survey type studies using questionnaires. Consider the use of this term”

**Author’s responses:** Thank you! We used the term response rate to refer to the incomplete records. Nevertheless, we accept the direction and prefer to remove in the main document (Please see Line 47).

2. **INTRODUCTION part:**

- **Comment #1:** “Line 74 is ambiguous, consider revising the last phrase: ‘‘…mortality in the early days of ICU admission before the causes of death are recognized’’

**Author’s responses:** We have rephrased the sentence to be clearer (Line 73 & 74).

- **Comment #2:** “Ethics Approval number should be included in the manuscript.”

**Author’s responses:** We have included the reference number (Line 148).

- **Comment #3:** “Use of the term ‘’response rate’’ also requires revision. this normally applies to qualitative/survey-type studies and does not seem to apply to this study type.”

**Author’s responses:** Thanks for the suggestion. We have already addressed this comment above in the abstract part. We did necessary amendments to the main document (Refer to Line 158 & 159).

3. **METHODS section:**

- **Comment #4:** " Methods: study design needs revision and clarity.”

**Author’s responses:** This comment was already addressed above under the abstract part and was clearly defined in the first paragraph of the methods part as well (Kindly refer to Line 101-103).

4. **On the DISCUSSION section:**

- **Comment #1:** “Line 229: the word 'lately' should be later”

**Author’s responses:** Sentence rephrased and changes made (Line 244).

- **Comment:** “Line 281: samples recruited does not sound right, you cannot recruit samples.”

**Author’s responses:** The term “recruited” was replaced with “selected” (Line 297).

5. **Conclusion section:**

- **Comment:** “Does an association with late mortality necessarily imply protection from early mortality? the researchers also need to be able to clarify how severe disease with complications could be physiologically protective; what physiological or clinical management factors could explain this finding?”

**Author’s responses:** We are thankful to the point raised. It seems the word choice and way of expression we used makes it ambiguous. Therefore, we rephrased the statement so that it could be sound for interpretation of the findings (Line 302 & 303). In fact, we cannot draw strong inferences regarding the associations and might need to be supported and proved by future studies using our conclusions as a baseline. The possible justifications regarding the physiological and clinical link between the complications and early mortality were presented above in the discussion section (Kindly refer to 282-289).

- **Comment:** “Line 289: avoid using the word 'halt': chronic diseases cannot easily be halted, their course tends to be slowly progressive.”

**Author’s responses:** Thank you again! The term “halt” was replaced with “control” (Line 305).
